# Migration and Health: A Framework for 21st Century Policy-Making

**DOI:** 10.1371/journal.pmed.1001034

**Published:** 2011-05-24

**Authors:** Cathy Zimmerman, Ligia Kiss, Mazeda Hossain

**Affiliations:** Gender Violence & Health Centre, Social and Mathematical Epidemiology Group, Department of Global Health & Development, London School of Hygiene & Tropical Medicine, London, United Kingdom

## Abstract

In the introductory article to a six-part *PLoS Medicine* series on Migration & Health, series guest editors Cathy Zimmerman, Mazeda Hossain, and Ligia Kiss outline a migratory process framework that involves five phases: pre-departure, travel, destination, interception, and return.

**Summary Points**

- Migration is a global phenomenon that influences the health of individuals and populations.
- Policy-making on migration and health is conducted within sector silos that frequently have different goals. Population mobility is wholly compatible with health-promoting strategies for migrants if decision-makers coordinate across borders and policy sectors.
- Policies to protect migrant and public health will be most effective if they address the multiple phases of the migratory process, including pre-departure, travel, destination, interception, and return. Health intervention opportunities exist at each stage.
- This article forms the introduction to a *PLoS Medicine* series on Migration & Health, laying out a new framework for understanding the migratory process and the five phases of migration, which are discussed in depth in five subsequent articles.

**This is one article in a six-part** ***PLoS Medicine*** **series on Migration & Health.**

## Introduction

With an estimated 214 million people on the move internationally and approximately three-quarters of a billion people migrating within their own country, there can be little doubt that population mobility is among the leading policy issues of the 21st century [1]–[3]. Human migration is not a new phenomenon, but it has changed significantly in number and nature with the growth of globalization, including the ease of international transport and communication, the push and pull factors of shifting capital, effects of climate change, and periodic political upheaval, including armed conflict. As a result, migrant networks that facilitate mobility and circular migration, in particular, have expanded in unprecedented ways [4],[5]. Yet, there has not been commensurate development of coordinated policy approaches to address the health implications associated with modern migration. Internationally, policy-making on migration has generally been conducted from policy sector “silos” (e.g., international aid, security, immigration enforcement, trade, and labor) that rarely include the health sector and which often have different, if not incompatible, goals [6],[7]. As discussions on “global migration governance” and “global health governance” expand, it will be increasingly important for policy-makers to engage in cross-sector coordination and move beyond narrow protectionist policy approaches, such as migrant-screening, and the simplistic view of migration as a one-way trajectory [8].

Health policy-making in the context of migration has generally been viewed either in terms of its “threats” to public health or from a rights-based approach that focuses on health hazards faced by individual migrants and the associated service challenges [9]. The former lens dates back to medieval quarantine measures and prioritizes public health security and communicable disease control, relying heavily on monitoring and screening (e.g., tuberculosis, pandemic flu). The rights-based perspective is more recent and grounded in medical ethics. It recognizes migrants' special vulnerability to, for example, interpersonal and occupational hazards, social exclusion, and discrimination, and the importance of universal access and culturally competent health care services [10].

Although often framed as a “threat”, human mobility is not inherently risk-laden. However, poor policy coordination and contradictory policy goals, such as increasing foreign labor requirements while maintaining restrictive rights for migrants, can exacerbate risk conditions related to migration and pose health challenges [11],[12].

**Figure 1:**
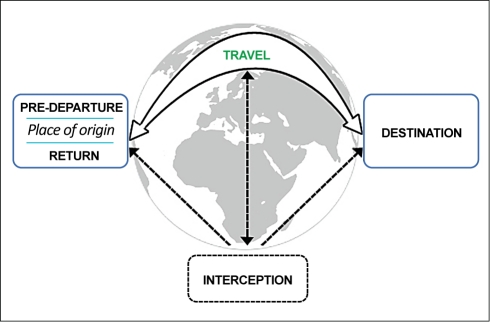
Migration phases framework.

This paper presents an introduction to the *PLoS Medicine* series on migration and health (http://www.ploscollections.org/migrationhealth). It lays out a migratory process framework (Figure 1) that highlights the multistaged and cumulative nature of the health risks and intervention opportunities that can occur throughout the migration process, and points to the potential benefits of policy-making that spans the full range of migratory movement. Five subsequent articles in the series discuss in-depth the health impacts and policy needs associated with the five phases of this migratory process: pre-departure, travel, destination, interception, and return.

## Global Estimates, Migrant Categories, and Gender

Theories and definitions of migration are diverse and include temporary and more permanent forms of human mobility that can occur for different purposes over long and short distances [13],[14]. Statistics on global migration are imprecise because of the diversity in definitions and due to the difficulty of counting irregular or undocumented migrants [15]. Table 1 presents some commonly used definitions and recent estimates for different mobile populations. Notably, internal migrants account for nearly four times as many individuals as international migrants. Among international migrants, it is estimated that nearly 50% of international movement is inter-regional [1].

**Table 1:** Definitions and estimates for mobile groups.

Migrant Category	Definition	Estimates	Data Year, Source
**International migrants**	Individuals who remain outside their usual country of residence for at least one year [3].	Estimated number of migrants at mid-year: 213,943,812 (Females: 49%; Males: 51%)	2009, UN Population Division [3]
**Internal migrants**	Individuals who move within the borders of a country, usually measured across regional, district, or municipal boundaries, resulting in a change of usual place of residence [1].	∼740 million	2000–2002, UNDP [1]
**Irregular migrants (or undocumented / illegal migrants)**	Individuals who enter a country, often in search of employment, without the required documents or permits, or who overstay the authorized length of stay in the country [58],[59].	∼20 to 30 million, comprising 10%–15% of the world's immigrant stock	2005, UN Population Division [3]
**Trafficked persons**	Individuals who are coerced, tricked, or forced into situations in which their bodies or labor are exploited, which may occur across international borders or within their own country [60].	Estimates unreliable	n/a
**International labor migrants (flow)**	Individuals engaged in a remunerated activity in a state of which he or she is not a national, including persons legally admitted as a migrant for employment [61].	Total: 27,390,884Total among countries with sex-disaggregated data: 3,037,335 (Females: 45%; Males: 55%)	2006, ILO [62]
**Internally displaced persons (IDPs)**	Individuals who have been forced to leave their homes or places of habitual residence, in particular, as a result of or in order to avoid the effects of armed conflict, situations of generalized violence, violations of human rights, or natural or man-made disasters, and who have not crossed an international border [63].	Total protected/assisted by UNHCR, including people in IDP-like situations: 15,628,057	End-2009, UNHCR [63]
**Refugees**	Individuals who, owing to a well-founded fear of being persecuted for reasons of race, religion, nationality, membership of a particular social group, or political opinion, are outside the country of their nationality, and are unable to, or owing to such fear, are unwilling to avail themselves of the protection of that country or return because of fear of persecution [64].	Refugees as a percentage of international migrants: 7.6%Total in refugee-like situations:10,396,540 (Females: 47%; Males: 53%)	2009, UN Population Division [3]End-2009, UNHCR [63]
**Asylum-seekers**	Individuals who have sought international protection and whose claims for refugee status have not yet been determined [63].	Applications made: 912,749	2009, UNHCR [63]
**Stateless persons**	Individuals not considered as citizens of any state under national law. Covers *de jure* and *de facto* stateless persons, including persons who are unable to establish their nationality. Stateless persons may or may not be migrants [63].	Total assisted by UNHCR: 208,869	2009, UNHCR [63]
**Tourists**	Individuals travelling to and staying in places outside their usual environment for not more than one consecutive year and whose main purpose of visit is other than work [65].	808 million (world)	2009, UN World Tourism Organization [65]
**International students**	Individuals admitted by a country other than their own, usually under special permits or visas, for the specific purpose of following a particular course of study in an accredited institution of the receiving country [66].	Total: 2,348,704Total among countries with sex-disaggregated data: 1,359,660 (Females: 45%; Males: 55%)	2007, UNESCO [67]

ILO, International Labour Organization; UNDP, United Nations Development Programme; UNESCO, United Nations Educational, Scientific and Cultural Organization; UNHCR, Office of the United Nations High Commissioner for Refugees.

For the past 50 years, there have been an approximately equal proportion of migrant women and men [2]. The motives and conditions of women's migration have changed markedly, with a growing number of women migrating independently for low-skill labor opportunities, rather than as spousal or family dependents. This has raised discussions about the “feminization of migration” [16]. At the same time as numerous women may gain greater independence and empowerment through migration, particular risks may arise such as, physical and sexual violence, including trafficking for forced sex work [17].

In migration statistics, it is not uncommon for different mobile groups and males and females to be classified together as “immigrants” or categorized solely by nationality [18],[19]. But people frequently fit into multiple categories or change their migration status over time and circumstances. From a public health policy perspective, an important dichotomy is between “documented” versus “irregular” and “forced” migrants. Individuals who travel via legal channels with required documentation, e.g., high-skilled laborers, are likely to encounter fewer health risks and have better service access than undocumented or “irregular” migrants [20].

## Phases of the Migratory Process and Health Considerations

Traditionally, policy-making has viewed migration as individual movement from point A to point B, generally focusing on permanent transnational resettlement. Yet, contemporary mobility is a much more complex process, more accurately viewed as a multistage cycle that can be entered into multiple times, in various ways, and may occur within or across national borders. Figure 1 depicts a migratory process model with five phases: pre-departure, travel, destination, interception (affecting a minority of migrants), and return [13],[21],[22]. This framework lends itself to more comprehensive and multinational policy-making. The five subsequent articles in the *PLoS Medicine* series will discuss in-depth these five phases, but here we provide a summary.

### Pre-Departure Phase

The pre-departure phase comprises the time before individuals leave from their place of origin. Factors that may influence health at this stage include biological characteristics, local chronic disease patterns and pathogens, environmental factors, and political and personal circumstances (e.g., human rights violations, interpersonal violence). Forced migrants are particularly likely to have experienced traumatic events at this stage, which may affect their psychological and physical health status throughout their journey. An individual's health status also frequently reflects health policies and the strength of the health sector, including health promotion, service quality, and access. Policy dialogues related to pre-departure locations have focused primarily on screening for communicable diseases [23],[24] and the depletion of health care professionals from resource-poor areas [25], with less attention to, for example, the health of the elderly and children who are left behind [26],[27]. Although this is a beneficial time to conduct health promotion and offer information to potential migrants about health in the destination location, there has been little collaboration between countries of origin and destination. However, several countries with large numbers of labor migrants have begun to develop programs to inform individuals about health risks and service rights [28],[29] and have implemented multilateral employment and social insurance schemes with recruitment agencies and with destination countries [30],[31] (examples in Table 2).

**Table 2:** Global migration and health policy instruments and agreements: Examples of international, regional, national, and internal policies.

Document	Institution	Brief Description
**International**		
**World Health Assembly (WHA)** **2005: International Health Regulations (IHR)** [68];**2007: Workers' Health** [69];**2008: Health of Migrants** [70]	World Health Organization (WHO)	**Purpose:** Decision-making body of WHO.**Specific health content:** WHA 2005 creates a public health response to prevent international spread of disease, avoiding unnecessary interference with international traffic and trade.WHA 2007 endorses global plan for full coverage of all workers' health.WHA 2008 promotes migrant-sensitive health policies.**Implementation and limitations:** WHA National IHR Focal Points established. Critiqued for narrow disease focus and concern for lack of states' political will [71].
**International Convention on the Protection of the Rights of All Migrant Workers and Members of their Families** [72]	United Nations convention	**Purpose:** Protects rights of migrants and family members, including protection against violence, injury, threats, arbitrary arrest or detention, and collective expulsion.**Specific health content:** Rights of migrants to receive urgently required medical care on the basis of equality of treatment with nationals of the state concerned.**Implementation or limitations:** Requires states to ensure that migrants have same rights as nationals to social and health services, provided that migrants meet requirements of national schemes.
**Regional**		
**MERCOSUR Multilateral Social Security Agreement** [73],[74]	Common Market of the South (MERCOSUR)	**Purpose:** Provides right to social security for persons working in member states (Argentina, Brazil, Paraguay, and Uruguay) and for their family members, ensuring same rights and obligations as nationals.**Specific health content:** Free care through the public health care network in the destination country for temporarily displaced workers and their dependents, if authorized by origin country.**Implementation or limitation:** Poor knowledge of the law may leave access open to local-level interpretation [75].
**Proposal for a Directive of the European Parliament and of the Council on the application of patients' rights in cross-border health care** [76],[77]	European Commission (27 member states)	**Purpose:** Guarantees quality and security in health services for cross-border health care and clarifies entitlements and limits of patients of member states.**Health content:** Entitles citizens to be reimbursed up to the cost of same or similar treatment in their national health system if the person is entitled to treatment in their country of affiliation, with certain health care requiring pre-authorization.**Implementation or limitations:** National contact points to report on standards and provide information to patients. States providing treatment may restrict access where justified.
**National**		
**Migrant Workers and Overseas Filipinos Act/ Philippine Overseas Employment Administration (POEA)** [78]–[80]	Philippines	**Purpose:** Assures rights of Overseas Philippine Workers (OFWs); guarantees deployment to countries ensuring protection, banning deployment if necessary; supports legal or unauthorized OFWs; stiff penalties for illegal recruiters and free legal assistance for victims, repatriation, and reintegration services.**Specific health content:** Mandates compulsory insurance cover for departing OFWs; requires licensed recruitment agencies or foreign employers to pay for insurance coverage (accidental or natural death, permanent disablement, repatriation cost, subsistence allowance, settlement claims, compassionate visit, medical evacuation, and medical repatriation) at no cost to the worker.**Implementation or limitations:** Establishes Migrant Workers and Other Overseas Filipinos Resource Centers in countries where there are large numbers of Filipinos, Legal Assistant for Migrant Workers Affairs (now the Office of the Undersecretary of Migrant Workers Affairs), and the Legal Assistance Fund.
**The ** ***hukou*** ** system** [81],[82]	China	**Purpose:** An official residence status that restricts Chinese citizens' access to public services to their place of birth.**Specific health content:** Internal migrants often do not qualify for public medical insurance and health assistance in a new or temporary place of residence.**Implementation and limitations:** The transferability of the *hukou* rights of residence from rural areas to cities is extremely difficult, as a result, few migrants can access public health services [82].

### Travel Phase

The travel phase encompasses the period when individuals are between their place of origin and a destination or an interception location. This phase might include multiple “transit” locations where individuals stop for short or long periods. From a global public health perspective, this is the stage during which pathogens may be carried across different zones of disease prevalence and initiate changes in international and local transmissible disease epidemiology. Travel restrictions have been a focus of attention after the recent outbreaks of pandemic influenza, even if there is limited evidence about their effectiveness [32],[33]. Especially for irregular migrants, health influences during this time are closely related to the mode of transport and circumstances of travel, such as journeys via flimsy boats or closed containers [34]. There are regular reports of Mexican migrants who die from heat exposure on treks across the desert towards the United States, or Burmese refugees fleeing through malaria-endemic areas [35],[36]. In cases of human trafficking, this phase is generally the time when criminal acts begin, such as illegal border crossings, kidnapping, and, for women and children, sexual violence. Evidence on health promotion programs at border or transit locations for migrants is scant. However, several health education and support initiatives have been established, for example, in US–Mexico border towns [37].

### Destination Phase

The destination phase is when individuals settle either temporarily or long-term in their intended location. A majority of migration health research and policy attention has focused on this phase, usually describing issues in high-income and migrant-receiving countries and frequently investigating specific diseases, certain ethnic groups, or “the healthy migrant effect” [38]. However, greater attention is required for non-communicable diseases, mental health, and socioeconomic influences on health. Risk behaviors among migrants appear to change when they are in new settings such as when Japanese migrants to the US showed that as cultural adaptation became more pronounced, the risk of coronary heart disease began to match that of the host population [39]. Mental health outcomes often appear worse for migrants, displaced populations, and refugees than for native-born populations [40]. Migrant women may be at greater risk of reproductive health problems and poor pregnancy outcomes, such as pregnancy complications, neonatal morbidity, and infant mortality [41]. Asylum-seekers with temporary protection tend to have poorer mental health than refugees who have permanent residency [42] and similarly, low-skilled migrant laborers, especially those with irregular status, are at high risk of injury and illness [43].

### Interception Phase

The interception phase applies to a small but particularly at-risk portion of the migrating population. This phase is characterized by situations of temporary detention or interim residence and is primarily relevant for forced migrants (e.g., asylum-seekers, refugees, displaced populations, trafficked persons) or irregular migrants, such as undocumented workers. Interception strategies for international migrants or displaced persons are frequently linked to immigration control policies and often have negative or punitive implications. Immigration detention centers or refugee camps often have deleterious effects on mental or physical health and are commonly sites of human rights abuses. There are clear associations between the length of detention and severity of mental disorders, especially for individuals with prior exposure to traumatic events, which is common among forced migrants. To date, few policy-level mandates have incorporated explicit measures to detect or prevent psychological morbidity in detention situations [44],[45]. In addition, detention conditions may be unhygienic or unsafe (particularly for women) [46],[47]. In high-resource settings, medical care for migrants in detention may be more advanced than in an individual's home country, but poorer compared to services available to the host population due to policies that, either by design or neglect, permit unequal treatment of migrants. Complex humanitarian emergency responses may be associated with the emergence of public health hazards by linking populations with disparate disease prevalence, but may also give rise to health-promoting measures, such as access to modern medical interventions and social services [48] and targeted prevention or treatment programs [49].

### Return Phase

The return phase is when individuals go back to their place of origin, either temporarily or to resettle indefinitely or permanently. In this phase, vulnerable migrants may experience the cumulative toll that migration exposures have taken on their physical and psychological well-being. In some settings, returning migrants, especially those who move from rural to urban areas, may be responsible for introducing new pathogens or increasing the prevalence of infections among the local population [50]. Individuals returning to low-resource settings with life-threatening, disabling, or chronic health concerns that require ongoing or high-tech treatment, such as cancer, diabetes, or HIV, may have difficulty identifying or paying for adequate care. People who return after suffering serious abuse, such as trafficked persons or war-affected refugees, may sustain high levels of distress or psychiatric morbidity [51],[52]. Practices related to the repatriation of individuals with life-threatening conditions do not always fully adhere to human rights principles and can put returnees at risk of long-term morbidity or mortality [53],[54]. Particularly in post-conflict situations when refugees are resettled to locations that have been ravaged by war, highly vulnerable individuals are likely to encounter a dearth of necessary services [55]. Many labor migrants, however, may return with reasonable remuneration and remittances that help them afford a healthier lifestyle and better health care for themselves and their family. There is a need for bilateral or regional agreements to support the portability of health care benefits, especially when healthy migrants contribute to wealthy countries and return unwell or to retire and require significant care from their home country's health system [56].

## Migration Health Policy Standards and Instruments

Table 2 presents examples of international instruments and regional and national legislation or policies related to health and migration. At the international level, the 61st World Health Assembly adopted a resolution that encouraged states to develop migrant-sensitive health policies and practices. The selected regional and national examples indicate the somewhat disjointed, sometimes conflicting, nature of migration health policy-making, as well as important gaps [57]. For instance, migrant health insurance schemes may be encumbered by restrictive immigration legislation or exclude undocumented migrants and migrants' family members from coverage. Similarly, regional agreements or national plans may promote economic cooperation through labor migration, but may not include portable health benefits. In practice, responsibility for fair health policies for migrants still lies within each nation state. And, even where multilateral agreements exist, their implementation does not automatically translate into universal, equal health opportunities for migrants.

## Conclusions

If internal and international migrants compris`ed a nation, it would be the third most populous country in the world, just after China and India. Yet, attention to the health of migrants is still limited. Where migration health policies exist, they operate primarily in isolation at national levels and cover only fragmented snapshots of people's movement, with few binding regional or global health protection agreements to respond to the true scope of contemporary migration [7],[8].

Moreover, the chasm between practice and policy—those providing health services to migrants versus those making policies about migrants' entitlements—is increasingly evident. At the same time that clinicians are treating more diverse migrant groups, policy-makers are attempting to implement restrictive or exclusive immigration-related health policies that contradict public health needs and undermine medical ethics that operate on the ground.

Policies that respond to the diversity of migrant groups and their differential health risks and service access must be developed and implemented. Moreover, to make real advances in the protection of both individual and public health, interventions must target each stage of the migration process and reach across borders. Services should be based on human rights principles that foster available and accessible care for individual migrants.

Migration policy-making is wholly compatible with health-promoting strategies for migrants. As globalization appears to be irreversibly linked to population mobility and individuals have proven that they will continue to migrate and re-migrate, it is time for decision-makers from the migration and health sectors to sit at the same table with policy-makers from other sectors, such as development, humanitarian aid, human rights, and labor, to make migration safe and healthy for all.
